# Effects of Thyroid Dysfunction on Angiogenesis During Wound Healing and Skin Repair: A Systematic Review

**DOI:** 10.7759/cureus.93343

**Published:** 2025-09-27

**Authors:** Alexandra Quadrozzi, Harvey N Mayrovitz

**Affiliations:** 1 Osteopathic Medical School, Nova Southeastern University Dr. Kiran C. Patel College of Osteopathic Medicine, Fort Lauderdale, USA; 2 Medical Education, Nova Southeastern University Dr. Kiran C. Patel College of Allopathic Medicine, Davie, USA

**Keywords:** angiogenesis, endostatin, hyperthyroid, hypothyroid, skin wounds, thyroid dysfunction, vegf, wound healing

## Abstract

Thyroid hormones (THs) play an important role in regulating cellular metabolism and tissue homeostasis, particularly influencing angiogenesis, an essential component of wound healing. This systematic review evaluates the current evidence on how thyroid dysfunction, hypothyroidism and hyperthyroidism, impacts angiogenic processes during cutaneous wound repair. A comprehensive search across PubMed, Web of Science, and Scopus identified 61 candidate studies. After applying inclusion criteria focused on original research evaluating TH status and angiogenesis in skin wound models, 26 high-quality studies were included. Evidence demonstrates that hypothyroidism leads to diminished angiogenic activity, delayed re-epithelialization, and decreased expression of vascular markers such as vascular endothelial growth factor (VEGF) and hypoxia-inducible factor-1α (HIF-1α). Conversely, hyperthyroid conditions showed enhanced neovascularization, although sometimes with dysregulated or excessive remodeling. Emerging experimental therapies, including thyroxine-impregnated biomaterials, nanofiber delivery systems, and stem cell-derived exosomes, also revealed promising angiogenic effects when modulated by THs. This review demonstrates the importance of thyroid status in wound care and highlights therapeutic opportunities for endocrine-modulated vascular repair.

## Introduction and background

Thyroid hormones (THs) are essential regulators of cellular growth, differentiation, and metabolic homeostasis, and they exert significant effects on the vascular system and the coordinated sequence of events involved in wound healing. The primary hormones, thyroxine (T4) and triiodothyronine (T3), play a central role in angiogenesis, an indispensable process in tissue repair, by promoting endothelial cell proliferation, directed migration, and capillary network formation [1,2]. Because angiogenesis is critical for delivering oxygen, nutrients, and reparative cells to injured tissues, disturbances in TH levels, such as those occurring in hypothyroidism or hyperthyroidism, can profoundly influence both the rate and the quality of wound healing [3,4].

Experimental and clinical studies have shown that THs regulate angiogenesis through two major and complementary mechanisms. Non-genomic signaling occurs through activation of the integrin alpha-V beta-3 (αvβ3) receptor, which rapidly initiates intracellular cascades such as mitogen-activated protein kinase/extracellular signal-regulated kinase (MAPK/ERK) and phosphoinositide 3-kinase/protein kinase B (PI3K/Akt) [1]. Genomic regulation is mediated by nuclear TH receptors, which control the transcription of genes essential for vascular growth and remodeling [1,5]. In hypothyroidism, diminished stimulation of these pathways leads to impaired angiogenesis, delayed re-epithelialization, reduced granulation tissue formation, and prolonged wound closure [4,6]. By contrast, restoration of TH levels through systemic replacement or localized application has been shown to activate endothelial cells, promote neovascularization, and accelerate tissue repair in both in vivo and ex vivo models [6-8].

Given the essential role of angiogenesis in wound repair, a thorough understanding of how THs influence this process has important therapeutic implications. Advances in biomaterials and regenerative medicine have expanded the possibilities for targeted delivery systems, including hormone-infused scaffolds [9,10], electrospun nanofiber dressings [10], platelet-rich plasma formulations [11], and stem cell-based therapies [12,13]. These strategies aim to produce sustained local pro-angiogenic effects while minimizing systemic exposure, making them particularly promising for wounds with compromised vascularization [9,10,14,15]. 

This review evaluates evidence from cellular, animal, and human studies to assess the impact of TH status on angiogenesis during wound healing. It also examines the molecular mechanisms that underlie this relationship and considers emerging therapeutic approaches that harness the angiogenic potential of THs for clinical wound care.

## Review

Methods

Study Design and Reporting Framework

This systematic review was conducted following the Preferred Reporting Items for Systematic Reviews and Meta-Analyses (PRISMA) 2020 guidelines [16,17]. The review protocol was designed with adherence to a structured approach using the Synthesis Without Meta-analysis (SWiM) reporting framework [16]. SWiM provides structured guidance when statistical pooling is inappropriate, ensuring that heterogeneity is addressed and findings are presented in a consistent and reproducible manner [16].

*Eligibility Criteria* 

To ensure relevance and quality, studies were included if they met the following criteria: (1) original peer-reviewed research articles written in English, (2) investigations involving either in vivo or in vitro models of skin wound healing, (3) evaluation of TH dysfunction (hypothyroidism, hyperthyroidism, or exogenous hormone administration), and (4) quantification of angiogenic outcomes such as vascular endothelial growth factor (VEGF) expression, hypoxia-inducible factor 1-alpha (HIF-1α) activity, endothelial cell density, or capillary proliferation. Exclusion criteria encompassed reviews, editorials, case reports, studies focused on non-cutaneous tissues (e.g., cardiac or neural angiogenesis), and articles lacking quantifiable angiogenesis-related endpoints.

Search Strategy

A comprehensive search was performed across PubMed, Web of Science, and Scopus covering the period from August 1999 to September 2025. The start date was chosen to capture studies conducted during a period when molecular and cellular investigations of angiogenesis and wound healing became methodologically standardized and when TH-related pathways began to be consistently examined in the biomedical literature. The search strategy combined Boolean operators and keywords: (“thyroid hormone” OR hyperthyroid OR hypothyroid) AND “angiogenesis” AND wound. Manual screening of reference lists and citation tracking supplemented the database search to ensure inclusion of all relevant studies examining the relationship between thyroid status, angiogenesis, and cutaneous wound healing.

Study Selection and Data Extraction 

A total of 61 studies were initially retrieved based on the search criteria. After title and abstract screening, followed by full-text review, 26 studies were selected for inclusion. Data extraction was conducted using a standardized form, capturing details such as author(s), year of publication, study model (animal, human, or cell culture), type of thyroid dysfunction examined, methods for assessing angiogenesis (e.g., immunohistochemistry, ELISA, and qPCR), and primary findings related to vascularization and wound healing.

Quality and Bias Assessment 

Quality and risk of bias were independently evaluated by two reviewers. For animal studies, the SYRCLE’s Risk of Bias Tool, adapted from the Cochrane tool, was utilized, assessing domains such as selection, performance, and detection bias [18]. For human observational studies, the Newcastle-Ottawa Scale was employed, evaluating cohort selection, comparability, and outcome assessment [19]. Discrepancies were resolved through consensus or consultation with a third reviewer. Only studies scoring moderate to high quality were retained in the final synthesis.

Data Synthesis and Analysis

Studies were thematically grouped by thyroid status (hypothyroidism vs. hyperthyroidism) and wound healing model (in vivo, ex vivo, or clinical). A structured narrative comparison was used to evaluate consistencies and differences across studies. Hypothyroid models consistently showed impaired angiogenesis, whereas hyperthyroid models demonstrated enhanced neovascularization. Variability between studies was largely attributable to methodological differences and model selection. Given the number of eligible studies and diverse outcome measures, subgroup or sensitivity analyses were not possible; however, potential sources of heterogeneity were considered in interpretation to ensure balanced conclusions.

Characteristics of the Selected Studies

**Figure 1:**
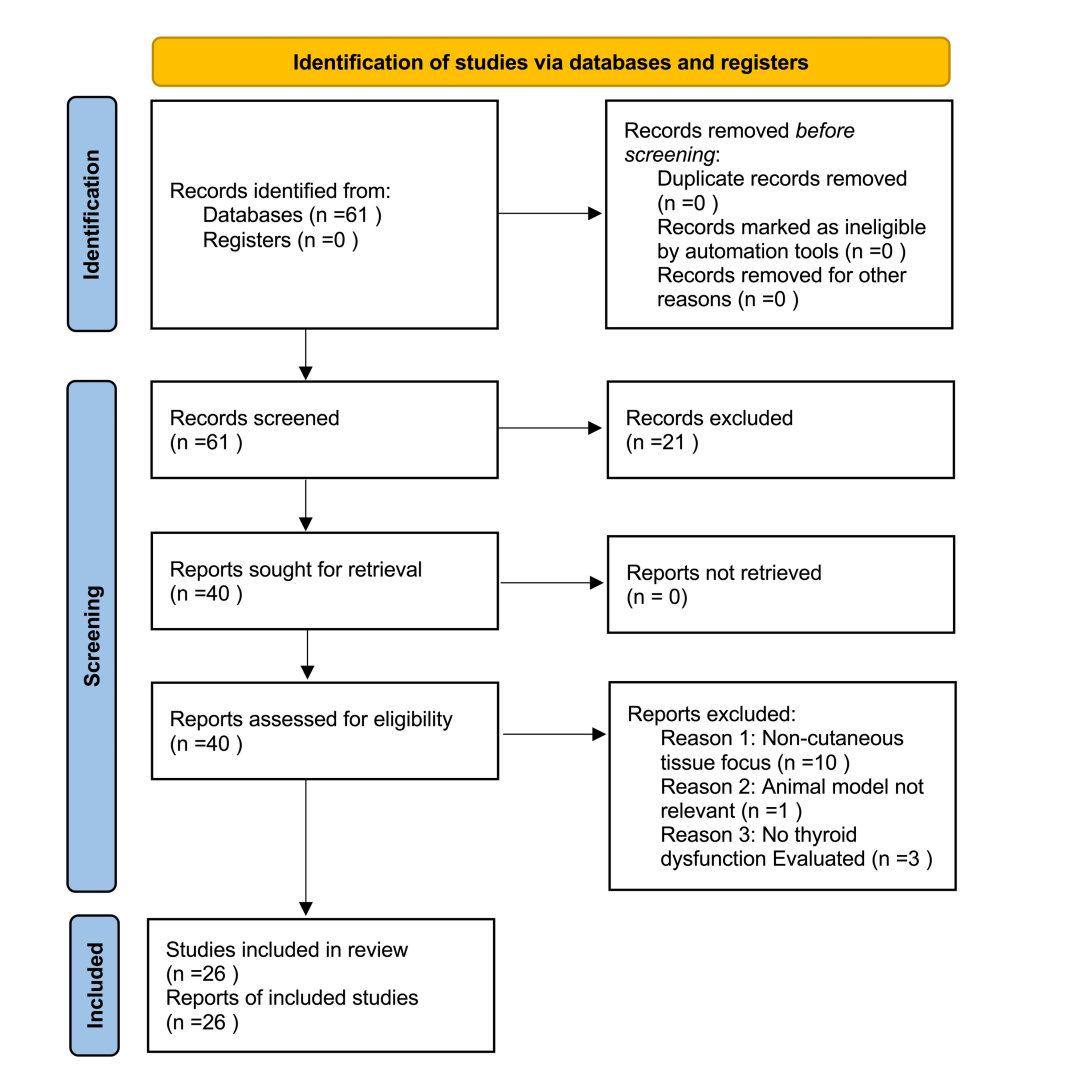
The Preferred Reporting Items for Systematic Reviews and Meta-Analyses (PRISMA) flowchart represents the study selection process.

A total of 61 records were identified through database searching, with no additional records from registers. After the initial screening of titles and abstracts, 21 studies were excluded, leaving 40 full-text articles sought for retrieval. All 40 reports were successfully obtained and assessed for eligibility. Following a detailed review, 14 studies were excluded for reasons including the use of non-cutaneous tissue models (n = 10), non-relevant animal models (n = 1), and the absence of thyroid dysfunction evaluation (n = 3). Ultimately, 26 studies met the inclusion criteria and were incorporated into this systematic review. The complete selection process is outlined in Figure 1.

Study Selection Process

Table 1 presents a comprehensive summary of the 26 studies included in this systematic review, including study design, model or population, thyroid dysfunction or intervention, and angiogenic outcomes. Although methodologies and scopes varied, all studies examined the influence of thyroid dysfunction on angiogenesis. Interventions ranged from the administration of triiodothyronine (T3) or thyroxine (T4) to experimental strategies assessing molecular signaling pathways, with outcomes reported through diverse markers of neovascularization and wound repair. This table served as the foundation for the thematic synthesis, allowing studies to be grouped by thyroid status, wound healing model, and angiogenic marker to facilitate structured narrative comparison.

**Table 1:** Characteristics of the 26 studies included in the systematic review This table summarizes the studies evaluating thyroid dysfunction, thyroid hormone interventions, and angiogenesis in wound healing. Reference indicates the numerical order of references. First Author, Year lists the first author and year of publication. Model / Population describes the experimental system, animal model, ex vivo tissue, or patient cohort. Thyroid Dysfunction / Intervention specifies the thyroid status (e.g., hypothyroidism, hyperthyroidism) or experimental treatment (e.g., topical T3/T4, scaffold delivery). Angiogenesis Marker / Outcome denotes the primary vascular or healing outcomes, such as VEGF expression, re-epithelialization, wound contraction, or complication rates. Abbreviations: integrin αvβ3, integrin alpha-V beta-3; MAPK, mitogen-activated protein kinase; TH, thyroid hormone; HMOX1-MSC, heme oxygenase-1-mesenchymal stem cell; PDGR-Akt, platelet-derived growth factor-protein kinase B; miRNA, microRNA; αvβ3/PKD/HDAC5, integrin alpha-V beta-3/protein kinase D/histone deacetylase 5; T3, triiodothyronine; T4, thyroxine; VEGF, vascular endothelial growth factor

First author, year	Model / population	Thyroid dysfunction / intervention	Angiogenesis marker / outcome
Bergh et al., 2005 [1]	In vitro (integrin αvβ3, endothelial cells)	Thyroid hormone binding to the integrin receptor	MAPK pathway activation, angiogenesis induction
Luidens et al., 2010 [2]	Review	Thyroid hormone and angiogenesis	Conceptual link between TH and angiogenesis
Kucharz et al., 2003 [3]	Human serum (hyper/hypothyroid patients)	Hypothyroidism and hyperthyroidism	Circulating endostatin levels
Rosko et al., 2018 [4]	Clinical (laryngectomy patients)	Hypothyroidism	Wound complications, healing outcomes
Davis et al., 2018 [5]	Review	Nongenomic thyroid hormone action	Mechanisms of TH signaling
Zhang et al., 2019 [6]	Ex vivo human skin	T4 topical/ex vivo	Enhanced re-epithelialization, angiogenesis
Safer et al., 2005 [7]	Mouse model	Topical T3	Accelerated wound healing, angiogenesis
Post et al., 2021 [8]	Ex vivo human skin (pathological conditions)	T4 supplementation	Restored re-epithelialization and angiogenesis
Badar et al., 2023 [9]	Alginate dressing (T4)	T4 alginate dressings	Angiogenesis stimulation
Satish & Korrapati et al., 2019 [10]	Nanofiber scaffold (T3 delivery)	T3 nanofiber	Sustained T3 release, angiogenesis
Ni et al., 2021 [11]	Rat skin defect model	Stem cells + PRP	Enhanced wound healing and angiogenesis
Cheng et al., 2024 [12]	Stem cell-derived exosomes	HMOX1-MSC exosomes	Angiogenesis, fibroblast function
Zeng et al., 2022 [13]	Stem cell spheroids	Reversible porous hydrogel	Wound healing, angiogenesis
Satish et al., 2019 [14]	Scaffold biomaterial (T3 delivery)	T3 scaffold	Angiogenesis in wound therapy
Waris et al., 2022 [15]	Cotton wound dressing (T4/heparin)	T4 + heparin dressings	Pro-angiogenic wound healing
Huang et al., 2022 [20]	Mouse cornea	Hypothyroidism	Delayed corneal healing
Chen et al., 2012 [21]	Hypothyroid mice (adult heart)	Thyroid hormone (systemic)	Sprouting angiogenesis via PDGR-Akt pathway
Arndt et al., 2018 [22]	Keratinocytes, fibroblasts, endothelial cells	Cold Atmospheric plasma (CAP)	Activation of angiogenesis-related molecules; improved wound angiogenesis (autocrine & paracrine)
Davis et al., 2009 [23]	Review	Thyroid hormone-induced angiogenesis	Angiogenesis induction
Paraguassu et al., 2014 [24]	Rat hypothyroid model	Hypothyroidism	Wound contraction, angiogenesis
Lin et al., 2018 [25]	Liver cancer cells	T3-induced angiogenesis	Angiogenesis in liver cancer
Ruiz-Llorente et al., 2018 [26]	Human skin, miRNA expression	Thyroid hormone receptors & miRNAs	Regulation of angiogenesis-related miRNAs
Ichiki et al., 2016 [27]	Review	Thyroid hormone vascular remodeling	Vascular remodeling, angiogenesis
Zinder et al., 2019 [28]	Review (Vitamin A)	Vitamin A, thyroid link	Role of vitamin A in angiogenesis
Tarameshloo et al., 2012 [29]	Rat wound model	T3 + Aloe vera gel	Accelerated healing, angiogenesis
Liu et al., 2014 [30]	In vitro, endothelial cells	T3 signaling pathway	Integrin αvβ3/PKD/HDAC5 pathway

Hypothyroidism

Clinical Evidence

A substantial body of research links hypothyroidism to impaired wound healing, with one of the most critical mechanisms being disruption of angiogenesis. Angiogenesis is essential during the proliferative phase of wound repair, as it ensures adequate oxygen delivery, nutrient transport, and recruitment of cells required for tissue regeneration. In hypothyroidism, this process is significantly impaired, consistent with the generalized metabolic slowdown seen in the condition [20,21].

Patients with hypothyroidism have elevated serum levels of endostatin, a potent endogenous inhibitor of angiogenesis, when compared to euthyroid individuals [3]. Endostatin is generated through cleavage of collagen XVIII and inhibits endothelial cell proliferation, migration, and capillary tube formation, producing a systemic environment that restricts new vessel growth [3,22]. Persistent elevation of endostatin shifts the balance away from pro-angiogenic activity, leaving the wound bed underperfused with blood and unable to efficiently progress through the stages of repair.

Clinically, this can manifest as prolonged inflammation, delayed granulation tissue maturation, and incomplete epithelialization [4]. Persistent hypoxia within the wound bed further impairs fibroblast function and extracellular matrix deposition, both of which are highly dependent on adequate vascular supply [12,23]. While large-scale clinical trials directly linking hypothyroidism to delayed wound healing remain limited, the consistent demonstration of elevated anti-angiogenic mediators, along with well-documented structural and metabolic tissue changes, supports a strong biological basis for this association. 

Animal Models

Controlled animal studies reinforce and expand upon clinical observations. Thyroidectomy-induced murine models, which mimic hypothyroid conditions, consistently show delayed re-epithelialization and reduced neovascularization compared to euthyroid controls [6,24]. Immunohistochemical analysis demonstrates markedly fewer proliferating endothelial cells, decreased VEGF expression, and disorganized stromal architecture [6].

Vascular impairment under hypothyroid conditions is further characterized by deficits in angiogenic signaling and significantly reduced collagen deposition [24]. Collectively, findings suggest that hypothyroidism disrupts the balance between pro-angiogenic factors such as VEGF and fibroblast growth factor 2 (FGF2) and anti-angiogenic mediators such as endostatin. This imbalance results in a poorly vascularized wound environment, limiting oxygen delivery, slowing keratinocyte migration, and delaying the transition from inflammation to proliferation.

Ex Vivo Human Skin Studies

Ex vivo human skin studies help clarify the direct impact of hypothyroidism on wound repair while removing systemic confounders. Skin explants maintained under hypothyroid-simulating conditions show reduced microvessel density, slower keratinocyte migration, and decreased keratinocyte proliferation [6]. Morphometric analysis reveals thinner epidermal layers and smaller volumes of granulation tissue compared to euthyroid controls.

Topical application of thyroxine to hypothyroid explants restores angiogenesis and epithelial coverage to near-normal levels while enhancing endothelial proliferation and overall tissue regeneration [6,8]. This reversal strongly suggests that observed impairments are a direct result of TH deficiency. These findings support the potential value of targeted, localized TH therapy in correcting vascular and epithelial deficiencies in patients with hypothyroidism.

Histological and Molecular Findings

Histological studies of hypothyroid wounds reveal fewer and more widely spaced capillary loops, immature vessels with inadequate lumen formation, and reduced extracellular matrix deposition [6]. These structural abnormalities reflect deeper molecular defects that impair vascular development.

At the signaling level, hypothyroidism is associated with reduced activation of the integrin αvβ3 receptor, a critical mediator of the non-genomic effects of THs on endothelial cells [1,5]. This reduction limits MAPK/ERK and PI3K/Akt pathway activation, thereby impairing endothelial cell migration, proliferation, and survival [5]. Elevated endostatin further inhibits VEGF signaling, suppressing endothelial cell activation and hindering the development of stable vascular networks [3]. Together, these molecular disruptions produce angiogenesis that is both delayed and structurally deficient, prolonging tissue hypoxia and slowing wound closure.

Summary of Hypothyroid Effects

Hypothyroidism exerts a broad inhibitory influence on wound healing, with angiogenesis impairment at the center of its pathophysiology. It increases angiogenesis inhibitors such as endostatin, reduces activation of key endothelial pathways, and limits endothelial cell proliferation. The result is delayed granulation tissue formation, reduced capillary density, immature vascular networks, and incomplete epithelial regeneration.

Evidence from animal models, diabetic-hypothyroid models, and ex vivo human skin studies shows that these vascular deficits are reversible. Localized TH supplementation has been shown to restore angiogenesis, increase endothelial proliferation, and promote tissue repair [6,8]. This provides strong support for further investigation into targeted TH therapy for patients with hypothyroidism who experience chronic, slow-healing, or non-healing wounds.

Hyperthyroidism

Clinical Evidence

Hyperthyroidism creates a strongly pro-angiogenic systemic environment by reducing endogenous inhibitors of vascular growth and increasing the expression of pro-angiogenic growth factors. Patients with hyperthyroidism have significantly lower circulating levels of endostatin compared to euthyroid individuals [3]. Since endostatin normally suppresses endothelial cell proliferation, migration, and capillary tube formation, its reduction in hyperthyroid states removes an important inhibitory checkpoint and facilitates the rapid development of vascular networks.

Alongside reduced inhibitory signaling, hyperthyroidism is associated with elevated VEGF expression, which stimulates endothelial sprouting, remodeling, and tissue blood perfusion [25]. This biochemical profile suggests that in hyperthyroid patients, the angiogenic phase of wound healing may progress more rapidly, potentially shortening both the inflammatory and early proliferative phases. Increased vascularization improves oxygen delivery and nutrient transport, supporting earlier fibroblast activation, collagen deposition, and keratinocyte migration.

Histological studies from case-control investigations reveal denser vascular networks in tissue samples from hyperthyroid patients, although some of these vessels display incomplete pericyte coverage and increased permeability [4]. These findings suggest that while hyperthyroidism accelerates vascular formation, the resulting networks may be structurally immature. This raises important considerations about the stability and long-term performance of vessels formed under sustained TH excess.

Animal Models

Experimental models provide further insight into the mechanisms by which excess TH stimulates angiogenesis. In murine models of TH deficiency, administration of thyroxine not only restores angiogenesis to euthyroid levels but often exceeds them, producing a supraphysiologic vascular response characterized by the formation of a greater number of new blood vessels and increased vascular density beyond what is normally observed under physiologic conditions [5,6]. This heightened angiogenic activity is linked to the activation of platelet-derived growth factor (PDGF) within the PI3K/Akt pathway, which regulates endothelial cell proliferation, migration, and survival. 

These effects occur in conjunction with MAPK/ERK pathway activation, which also promotes robust endothelial cell expansion and capillary network formation. Histological examination of thyroxine-treated animals demonstrates markedly increased microvascular density, extensive branching of vessels, and greater numbers of perfused capillaries compared to controls [5].

In hyperthyroid rat models, wound closure occurs more quickly, and healed tissues display denser vascular networks [24]. However, incomplete pericyte coverage is frequently observed, suggesting that rapid vessel proliferation may outpace stabilization processes. Prolonged hyperthyroidism has also been associated with increased vascular permeability, which could influence long-term vessel integrity.

Cellular and Ex Vivo Studies

Cell culture experiments and ex vivo human tissue models demonstrate that THs enhance angiogenesis through both genomic and non-genomic pathways. In endothelial cells, thyroxine binds to the integrin αvβ3 receptor and rapidly activates MAPK/ERK signaling, stimulating proliferation, motility, and early network assembly [1]. These effects occur independently of nuclear TH receptors, allowing rapid modulation of endothelial cell activity without the delay associated with transcriptional regulation.

In ex vivo human skin models, topical application of triiodothyronine accelerates re-epithelialization, increases microvessel density, and promotes capillary maturation, even in samples from euthyroid individuals [7]. Histological analysis reveals organized vascular networks within the wound bed, supporting the idea that local TH exposure can directly enhance structural and functional vessel development.

When combined with other pro-regenerative agents such as platelet-rich plasma, triiodothyronine produces an amplified healing effect, likely due to synergistic activation of complementary angiogenic pathways [20]. These findings suggest that systemic hyperthyroidism may amplify these local mechanisms, leading to accelerated and widespread vascular growth during wound repair.

Histological and Molecular Findings

Histological evaluations of wounds in hyperthyroid conditions consistently show denser capillary networks, higher endothelial proliferation rates, and more extensive extracellular matrix remodeling compared to euthyroid controls [5,7]. These structural patterns are underpinned by persistent activation of integrin αvβ3-mediated signaling, which maintains high levels of MAPK/ERK and PI3K/Akt pathway activity.

This sustained stimulation increases VEGF and FGF2 expression, both of which drive vessel branching, maturation, and stabilization [25]. However, some newly formed vessels exhibit increased permeability and incomplete maturation, reflecting a potential mismatch between rapid endothelial expansion and slower processes of vessel stabilization, such as pericyte recruitment and extracellular matrix deposition [1].

While these networks can accelerate early wound closure by improving oxygen and nutrient delivery, their long-term stability remains uncertain. Incomplete stabilization could predispose tissues to future microvascular fragility, making this an important area for further investigation.

Summary of Hyperthyroid Effects

Hyperthyroidism enhances angiogenesis by reducing inhibitory signals, such as endostatin, and elevating growth factors, including VEGF and FGF2, thereby activating pro-angiogenic signaling cascades. These changes promote rapid endothelial proliferation, early network assembly, and accelerated wound closure. However, structural maturity may lag behind the pace of vessel formation, leading to increased permeability and potential instability of newly formed vascular networks.

Animal, cellular, and ex vivo studies all support the concept that hyperthyroidism fosters a highly pro-angiogenic environment. While this may be advantageous for early repair, the long-term durability and functionality of these vessels require further study to fully understand the implications of sustained TH excess on wound healing outcomes.

Molecular mechanisms of TH-mediated angiogenesis

THs, particularly T4 and T3, regulate angiogenesis through both rapid non-genomic pathways and slower genomic transcriptional programs. These mechanisms influence endothelial cell proliferation, migration, lumen formation, and the stabilization of newly formed vessels, all of which are critical for effective wound healing. Through this dual mode of action, THs ensure that developing tissues receive adequate oxygen, nutrients, and reparative cells at the appropriate stages of repair.

Alterations in TH status disrupt this balance. Hypothyroidism suppresses vascular growth by increasing angiogenesis inhibitors such as endostatin, downregulating key signaling cascades, and impairing endothelial survival. Hyperthyroidism, in contrast, promotes vascular growth through the expression of elevated pro-angiogenic factors, sustained activation of intracellular signaling pathways, and reduced inhibitory control. 

Integrin αvβ3-Mediated Non-Genomic Signaling

One of the most important rapid-acting mechanisms in TH-regulated angiogenesis is mediated by integrin αvβ3, which is highly expressed on endothelial cells during vessel formation. Both T4 and T3 bind to a specific extracellular site on integrin αvβ3, triggering the activation of MAPK/ERK1/2 and PI3K/Akt signaling cascades [1,5]. These cascades rapidly promote endothelial proliferation, migration, and early capillary network assembly.

In an ex vivo human skin wound model, topical application of T3 significantly increased microvessel density and accelerated re-epithelialization without relying on nuclear TH receptors [7]. This demonstrates that non-genomic signaling through integrin αvβ3 can produce immediate effects on endothelial activity, bypassing the slower genomic processes of transcription and translation.

In hyperthyroidism, persistently high levels of circulating THs maintain chronic activation of integrin αvβ3, sustaining a heightened angiogenic state throughout wound repair. In hypothyroidism, reduced hormone availability leaves integrin αvβ3 under-stimulated, limiting endothelial migration and proliferation, delaying new vessel formation, and slowing the overall repair process.

Genomic Regulation via Nuclear Receptors

In addition to rapid non-genomic effects, THs also influence angiogenesis through genomic pathways mediated by nuclear TH receptors, thyroid receptor alpha (TRα), and thyroid receptor beta (TRβ). When activated by T3 or T4, these receptors regulate the transcription of genes involved in endothelial proliferation, extracellular matrix remodeling, and vessel stabilization, including VEGF, FGFs, and angiopoietins [25,26].

Under hypothyroid conditions, reduced TH availability leads to significant downregulation of these angiogenic genes. Ex vivo studies on human skin have shown thinner epidermal layers, decreased granulation tissue, and reduced vascular density in hypothyroid conditions [6]. Restoration of T4 reverses these deficits, improving vascular architecture and epithelial coverage, highlighting the essential role of genomic signaling in wound repair.

In hyperthyroid states, genomic activation increases VEGF and FGF expression and upregulates endothelial nitric oxide synthase (eNOS), thereby enhancing vasodilation and tissue blood perfusion. This genomic effect complements the rapid non-genomic pathways, producing both an immediate and sustained pro-angiogenic environment.

PI3K/Akt and PDGF Signaling Pathways

The PI3K/Akt pathway is another central mediator of angiogenesis that is modulated by THs. In hypothyroid mouse models, administration of T4 stimulates PDGF-dependent PI3K/Akt activation, resulting in robust sprouting angiogenesis and improved endothelial survival [5]. This pathway also supports the maturation of vascular networks by promoting the recruitment of pericytes and the stabilization of the extracellular matrix.

In hyperthyroidism, prolonged activation of the PDGF-Akt signaling pathway enhances microvascular density and branching but can also lead to the formation of immature vessel structures if stabilization mechanisms do not keep pace. In hypothyroidism, insufficient activation of this pathway leads to fewer newly formed vessels and delays in the transition from early vascular sprouting to mature vascular networks.

Modulation of Angiogenesis Inhibitors

THs regulate angiogenesis not only by stimulating pro-growth pathways but also by modulating angiogenesis inhibitors. In hypothyroidism, elevated serum endostatin interferes with VEGF signaling, suppressing endothelial proliferation and migration [3]. The persistence of high endostatin levels prolongs wound hypoxia and slows the development of granulation tissue. In hyperthyroidism, endostatin levels are significantly lower, removing inhibitory constraints on angiogenesis. This facilitates rapid vessel formation but may also increase the risk of unstable or hyperpermeable capillary networks.

VEGF and FGF Upregulation

VEGF and FGF are critical for initiating and sustaining angiogenesis. VEGF stimulates endothelial proliferation and vascular permeability, while FGF contributes to extracellular matrix remodeling and vessel stabilization. TH activation of the MAPK/ERK pathway increases both VEGF and FGF expression [1,7]. In hyperthyroid states, high levels of VEGF and FGF support the development of dense and highly branched vascular networks, thereby accelerating wound closure. In hypothyroidism, reduced VEGF and FGF expression limits both the initiation and stabilization of new vessels, prolonging hypoxia and delaying repair [6,24].

Crosstalk Between Genomic and Non-genomic Pathways

There is functional crosstalk between genomic and non-genomic mechanisms. Integrin αvβ3 activation can lead to phosphorylation of nuclear TH receptors, enhancing their transcriptional activity [1,26]. Conversely, activation of genomic pathways can upregulate integrin expression, strengthening non-genomic signaling. This bidirectional reinforcement ensures that rapid endothelial activation is followed by sustained gene-driven remodeling, allowing early sprouting angiogenesis to mature into stable vascular networks.

HIF-1α Modulation

THs also influence HIF-1α, a key regulator of angiogenesis under low oxygen conditions. Hypothyroidism reduces HIF-1α stabilization, impairing the transcription of hypoxia-responsive genes such as VEGF [6]. Hyperthyroidism maintains or elevates HIF-1α levels, supporting vascular growth in hypoxic wound environments [25]. While enhanced HIF-1α activity in hyperthyroidism can accelerate oxygen delivery to tissues, persistent elevation may promote excessive or disorganized vascular proliferation. This highlights the need for balance in HIF-1α signaling to ensure effective but stable angiogenesis.

Therapeutic applications of THs in wound healing

THs play a key role in stimulating angiogenesis, supporting cellular proliferation, and promoting extracellular matrix remodeling, which together make them promising candidates for therapeutic interventions in wound healing. Both systemic and localized delivery approaches have demonstrated the ability to increase vascular density, accelerate re-epithelialization, and enhance tissue repair in experimental models [7,14,27,28]. By targeting the molecular pathways that regulate angiogenesis, TH-based therapies have the potential to correct the vascular deficits seen in hypothyroidism and further optimize the already heightened angiogenic activity observed in hyperthyroidism.

Topical TH Formulations

Topical application of THs offers a way to bypass systemic fluctuations and deliver high concentrations directly to the wound site. In an ex vivo human skin model, triiodothyronine treatment increased microvessel formation, enhanced keratinocyte proliferation, and accelerated epithelial closure [14]. Histological analysis revealed denser and more mature vascular networks in treated wounds compared to controls. This finding is consistent with local TH exposure activating angiogenic pathways within the wound bed. Animal studies have demonstrated similar effects. Topical thyroxine applied to full-thickness rat wounds improved granulation tissue formation, increased epithelial coverage, enhanced collagen fiber alignment, and strengthened wound tensile properties [7]. These improvements were linked to both genomic and non-genomic activation of endothelial cells, including increased VEGF expression and integrin αvβ3-mediated signaling.

Hormone-Impregnated Biomaterials and Advanced Delivery Systems

Incorporating THs into biocompatible scaffolds can allow for sustained local release while also protecting the wound from external contamination. Triiodothyronine-loaded alginate-gelatin-polyvinyl alcohol scaffolds have been shown to maintain steady hormone release in preclinical studies, producing robust angiogenesis, increased fibroblast proliferation, and well-organized extracellular matrix formation [14]. Another example is a cotton-based dressing containing thyroxine and heparin, which produced both pro-angiogenic and anti-thrombotic effects. This dressing increased neovascularization, reduced fibrin deposition, and accelerated epithelial closure when compared to standard gauze dressings [15]. The combination of a growth-promoting hormone with an anti-clotting agent may be particularly beneficial in wounds where microvascular thrombosis impairs healing.

Nanofiber Delivery Systems

Electrospun nanofiber scaffolds represent an advanced delivery platform that can provide prolonged and localized hormone exposure with minimal systemic absorption. Studies loading triiodothyronine or thyroxine into nanofibers have demonstrated that these systems sustain therapeutic hormone concentrations at the wound site for extended periods, continuously activating endothelial cells and promoting capillary maturation [10]. This type of sustained angiogenic signaling can accelerate wound closure while supporting vessel stabilization, thereby reducing the risk of immature or leaky vascular networks. Nanofiber-based systems may be particularly useful for patients in whom systemic TH supplementation is contraindicated due to the risk of inducing hyperthyroidism or other metabolic complications.

Combination Therapies

Combining THs with other pro-regenerative agents can enhance healing outcomes through synergistic mechanisms. In a rat wound model, thyroxine cream combined with aloe vera gel achieved the fastest re-epithelialization, the highest vascular density, and the most organized collagen structure compared to either treatment alone [29]. The combination of triiodothyronine with platelet-rich plasma improved vascular maturation and epithelialization in diabetic wound models, likely due to the integration of TH-mediated endothelial activation with the growth factor-rich environment provided by platelet-derived products [11,20]. Similarly, mesenchymal stem cell-derived exosomes enriched with THs promoted rapid granulation tissue development and the formation of well-organized vascular networks [30].

Clinical Translation and Considerations

Although the preclinical evidence supporting TH-based wound therapies is strong, translation into clinical practice requires careful consideration. Local delivery methods can minimize systemic effects, but some systemic absorption is possible, which may pose risks in patients with normal or elevated baseline thyroid function [3]. The patient’s thyroid status may also influence treatment efficacy and safety, as hypothyroidism is associated with elevated endostatin levels and hyperthyroidism is associated with reduced endostatin levels, each of which modifies angiogenic regulation. For this reason, individualized treatment planning is essential. Factors such as baseline thyroid function, wound type, and comorbid conditions should guide therapeutic decisions. Optimizing dosing schedules, selecting the most effective delivery platform, and monitoring for systemic effects will be critical in advancing TH-based wound therapies into routine practice.

Summary of Therapeutic Potential

Targeted delivery of THs through topical creams, hormone-loaded scaffolds, nanofiber systems, and combination regimens can enhance angiogenesis, improve granulation tissue formation, and accelerate epithelial regeneration [2,9,10,20,23]. These effects are mediated through multiple pathways, including integrin αvβ3 activation, VEGF and FGF upregulation, PDGF-Akt signaling, and modulation of angiogenesis inhibitors. While the preclinical results are compelling, large-scale clinical trials are necessary to determine the optimal formulations, dosages, and delivery schedules, as well as to ensure safety across diverse patient populations. If successfully translated, TH-based therapies could become a valuable component of advanced wound care, particularly for patients with impaired angiogenesis due to thyroid dysfunction.

## Conclusions

Thyroid status represents a crucial and modifiable determinant of wound healing outcomes by regulating angiogenesis, and localized TH therapy offers a promising translational avenue for clinical application. THs are not merely regulators of metabolism; they serve as potent modulators of angiogenesis and wound repair. Acting through both genomic and non-genomic mechanisms, T3 and T4 influence the entire spectrum of vascular regeneration, from endothelial activation and migration to vessel maturation and stabilization. Hypothyroidism impairs these processes, leading to delayed granulation tissue formation, incomplete epithelial coverage, and prolonged wound closure. By contrast, hyperthyroidism or targeted TH administration enhances pro-angiogenic signaling, growth factor production, and capillary sprouting. These opposing outcomes emphasize the importance of thyroid status in determining the pace and quality of tissue repair.

Evidence also suggests that localized TH delivery in the form of topical formulations, scaffolds, or nanofiber constructs can restore angiogenic activity and improve wound healing. Combination strategies that integrate THs with platelet-rich plasma or stem cell-based therapies demonstrate promising synergistic effects. Future investigations should aim to establish optimal dosing, refine delivery systems, and identify patient groups most likely to benefit from these interventions. It will also be essential to clarify how to balance rapid vascular growth with long-term vessel stability in order to ensure effective and safe application. If these challenges can be addressed, TH-based therapies have the potential to transform the management of chronic wounds, surgical recovery, and other angiogenesis-dependent conditions.
